# 

**Published:** 1917-02

**Authors:** 


CONTENTS.

ORIGINAL COMMUNICATIONS .......... 439
EDITORIAL .......... 453
SELECTIONS AND ABSTRACTS .......... 475
BOOK REVIEW .......... 467

